# Active inference, communication and hermeneutics^☆^

**DOI:** 10.1016/j.cortex.2015.03.025

**Published:** 2015-07

**Authors:** Karl J. Friston, Christopher D. Frith

**Affiliations:** The Wellcome Trust Centre for Neuroimaging, Institute of Neurology, UCL, United Kingdom

**Keywords:** Communication, Neuronal, Hermeneutics, Theory of mind, Active inference, Predictive coding, Bayesian, Synchronisation of chaos

## Abstract

Hermeneutics refers to interpretation and translation of text (typically ancient scriptures) but also applies to verbal and non-verbal communication. In a psychological setting it nicely frames the problem of inferring the intended content of a communication. In this paper, we offer a solution to the problem of *neural hermeneutics* based upon *active inference*. In active inference, action fulfils predictions about how we will behave (e.g., predicting we will speak). Crucially, these predictions can be used to predict both self and others – during speaking and listening respectively. Active inference mandates the suppression of prediction errors by updating an internal model that generates predictions – both at fast timescales (through *perceptual inference*) and slower timescales (through *perceptual learning*). If two agents adopt the same model, then – in principle – they can predict each other and minimise their mutual prediction errors. Heuristically, this ensures they are singing from the same hymn sheet. This paper builds upon recent work on active inference and communication to illustrate perceptual learning using simulated birdsongs. Our focus here is the neural hermeneutics implicit in learning, where communication facilitates long-term changes in generative models that are trying to predict each other. In other words, communication induces perceptual learning and enables others to (literally) change our minds and *vice versa*.

## 1 Introduction

The term hermeneutics refers to the art of interpreting written texts such as holy scriptures. The key problem for hermeneutics rests on developing criteria for deciding when an interpretation is correct. This problem is not restricted to the interpretation of ancient texts. When talking to you, I cannot access your mind to check whether my interpretation of what you have just said corresponds to what you meant. I can invent a coherent story or narrative, but I can never independently verify my interpretations (Frith & Wentzer, 2013). Nevertheless, people seem to understand each other most the time. How is this achieved? In this paper, we suggest the criteria for evaluating and updating my interpretation of your behaviour are exactly the same criteria that underlie action and perception in general; namely, the minimisation of prediction error or (variational) free energy.

In a companion paper (Friston and Frith, 2015), we considered communication in terms of inference about others, based on the notion that we model and predict our sensations – sensations that are generated by other agents like ourselves. This leads to a view of communication based on a generative model or narrative that is shared by agents who exchange sensory signals. Given a shared narrative, communication can then be cast as *turn taking* (Wilson & Wilson, 2005), by selectively attending and attenuating sensory information. Attending to exteroceptive sensations enables the shared narrative to predict the sensory input generated by another (while listening). Conversely, attenuating exteroceptive input enables one to articulate the narrative by realising proprioceptive predictions (while speaking). Using simulations, we demonstrated this turn taking by assuming that both agents possessed the same generative model. In this paper, we consider how and why generative models learned by agents – who exchange sensory signals – become the same (shared) model.

Our underlying premise is that we are trying to model the causes of our sensations – and adjust those models to maximise Bayesian model evidence or, equivalently, minimise surprise (Brown & Brün, 2012; Kilner, Friston, & Frith, 2007). This perspective on action and perception has broad explanatory power in several areas of cognitive neuroscience – and enjoys support from several lines of neuroanatomical and neurophysiological evidence (Egner & Summerfield, 2013; Rao & Ballard, 1999; Srinivasan, Laughlin, & Dubs, 1982). In communication and the interpretation of intent, the very notion of *theory of mind* speaks directly to inference, in the sense that theories make predictions that have to be tested against (sensory) data. Imagine two brains, each mandated to model the (external) states of the world causing sensory input. Now imagine that sensations can only be caused by (the action of) one brain on the other. This means that the first brain has to model the second. However, the second brain is modelling the first, which means the first brain must have a model of the second brain, which includes a model of the first – and so on *ad infinitum*. At first glance, the implicit infinite regress appears to preclude a veridical modelling of another's brain. However, this infinite regress dissolves if each brain models the sensations caused by itself and the other as being generated in the same way. In other words, if there is a shared narrative or dynamic that both brains subscribe to, then they can predict each other exactly – at least for short periods of time. This is the basic idea that we pursue in the context of active inference and predictive coding.

In our previous paper, we focused on the dynamical phenomena that emerge when two dynamical systems try to predict each other. Mathematically, this dynamical coupling is called *generalised synchrony* (aka synchronisation of chaos) (Barreto, Josic, Morales, Sander, & So, 2003; Hunt, Ott, & Yorke, 1997). Generalised synchrony was famously observed by Huygens in his studies of pendulum clocks – that synchronized themselves through the imperceptible motion of beams from which they were suspended (Huygens, 1673). This nicely illustrates the *action at a distance* among coupled dynamical systems. Put simply, generalised synchronisation means that knowing the state of one system (e.g., neuronal activity in the brain) means one can predict the another system (e.g., another's brain).

**Fig. 1:**
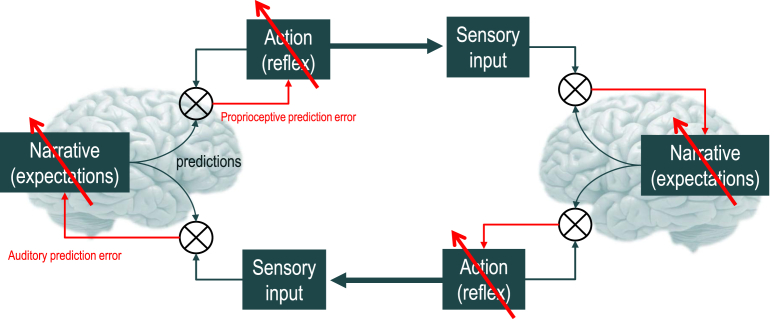
A predictive coding formulation of the Hermeneutic Circle: e.g., Speech Chain in the setting of language (Denes & Pinson, 1993). This schematic provides a simple example of neural hermeneutics in the form of a control system diagram. It is a simple example because the internal (generative) model predicting the behavioural consequences of action (of self and other) is the same. In other words, it is neither a model of my behaviour or your behaviour – but a model of our behaviour. When both agents adopt the same model, generalised synchronisation is guaranteed and prediction errors are minimised. The implicit architecture highlights the fact that the top-down predictions from a dynamical generative model (labelled Narrative) come in two flavours: exteroceptive predictions predicting the external consequences of action (c.f., corollary discharge) and proprioceptive predictions that predict the internal consequences of action (c.f., motor commands). These predictions are compared with sensory input to provide prediction errors. In control diagrams of this sort ⊗ denotes a comparator. Exteroceptive (e.g., auditory) prediction errors are used to update the generative model at various timescales to produce inference and learning. In contrast, the proprioceptive prediction errors drive classical reflexes to produce the predicted action. When the (dynamics of the) generative models in the two brains are identical, both exteroceptive and proprioceptive prediction errors are minimised and the dynamics will exhibit (generalised) synchrony. The red arrows denote learning or control by prediction errors that compare (descending) predictions with (ascending) sensations.

We will consider a special case of generalized synchronization; namely, *identical synchronization*, in which there is a one-to-one relationship between the states of two systems. Identical synchronisation emerges when the systems that are coupled are the same. In the context of active inference, this means the two generative models are identical. But why should two agents have the same generative model? The answer is rather obvious – when they share the same generative model they can predict each other more accurately and minimise their prediction errors or surprise. The key point here is that the same principle that leads to generalised synchrony also applies to the selection or learning of the model generating predictions. This learning is the focus of the current paper, which provides an illustrative proof of principle that the hermeneutic cycle can be closed by simply updating generative models and their predictions to minimise prediction errors. Crucially, these prediction errors can be computed without ever knowing the true state of another; thereby solving the problem of hermeneutics (see Fig. 1).

The treatment of communication in this paper is rather abstract and borrows mathematical concepts from dynamical systems theory. Although we will use birdsong as a vehicle to illustrate the ideas, we do not pretend this is a meaningful model of linguistic communication (or indeed songbirds). Rather, we try to understand the dynamic coordination of richly structured behaviours, such as singing and dancing, without ascribing any (semantic) meaning or syntax to sensory exchanges. Having said this, there is growing interest in applying the principles of predictive coding to language: e.g., (Arnal & Giraud, 2012; Hickok, 2013; Pickering & Clark, 2014; Wang, Mathalon, et al., 2014) – and understanding the algebra of dynamical systems in terms of communication; e.g., (Scott-Phillips & Blythe, 2013). Furthermore, predictive coding is starting to shed light on spectral asymmetries – in coupling within the auditory hierarchy – evident in electrophysiological studies of speech processing (Arnal, Wyart, & Giraud, 2011).

This paper comprises five sections. The first sections reprise the material in (Friston and Frith, 2015), which provides a brief review of active inference and predictive coding in communication. In the second section, we described the particular (birdsong) model used to illustrate communicative inference. This model has been used previously to illustrate several phenomena in perception; such as perceptual learning, repetition suppression, and the recognition of stimulus streams with deep hierarchical structure (Friston & Kiebel, 2009; Kiebel, Daunizeau, & Friston, 2008). In the third section, we provide a simple illustration of omission related responses – that are ubiquitous in neurophysiology and illustrate the basic nature of predictive coding. In the fourth section, we use this model to simulate two (identical) birds that are singing to themselves (and each other) and examine the conditions under which generalised synchrony emerges. In the final section, we repeat the simulations using two birds that start off with different generative models. We will see that perceptual learning produces a convergence of the two generative models over time – leading to the emergence of generalised synchrony and implicit communication. We offer this as a solution to the problem of hermeneutic inference that can be resolved by neuronally plausible schemes – with the single imperative to minimise prediction error or free energy.

## 2 Active inference and predictive coding

Recent advances in theoretical neuroscience have produced a paradigm shift in cognitive neuroscience. This shift is away from the brain as a passive filter of sensations – or an elaborate stimulus-response link – towards a view of the brain as an organ that generates hypotheses or fantasies (fantastic: from Greek *phantastikos*, the ability to create mental images, from *phantazesthai*), which are tested against sensory evidence (Gregory, 1968). This perspective dates back to the notion of unconscious inference (Helmholtz, 1866/1962) and has been formalised to cover deep or hierarchical Bayesian inference – about the causes of our sensations – and how these inferences induce beliefs, movement and behaviour (Clark, 2013; Dayan, Hinton, & Neal, 1995; Friston, Kilner, & Harrison, 2006; Hohwy, 2013; Lee & Mumford, 2003).

### 2.1 Predictive coding and the Bayesian brain

Modern formulations of the Bayesian brain – such as predictive coding – are now among the most popular explanations for neuronal message passing (Clark, 2013; Friston, 2008; Rao & Ballard, 1999; Srinivasan et al., 1982). Predictive coding is a biologically plausible process theory for which there is a considerable amount of anatomical and physiological evidence. In these schemes, neuronal representations – in higher levels of cortical hierarchies – generate predictions of representations in lower levels (Friston, 2008; Mumford, 1992; Rao & Ballard, 1999). These top-down predictions are compared with representations at the lower level to form a prediction error (usually associated with the activity of superficial pyramidal cells). The resulting mismatch signal is passed back up the hierarchy to update higher representations (associated with the activity of deep pyramidal cells). This recursive exchange of signals suppresses prediction error at each and every level to provide a hierarchical explanation for sensory inputs that enter at the lowest (sensory) level. In computational terms, neuronal activity encodes beliefs or probability distributions over states in the world that cause sensations (e.g., my visual sensations are caused by a *face*). The simplest encoding corresponds to representing the belief with the expected value or *expectation* of a (hidden) cause. These causes are referred to as *hidden* because they have to be inferred from their sensory consequences.

**Fig. 2:**
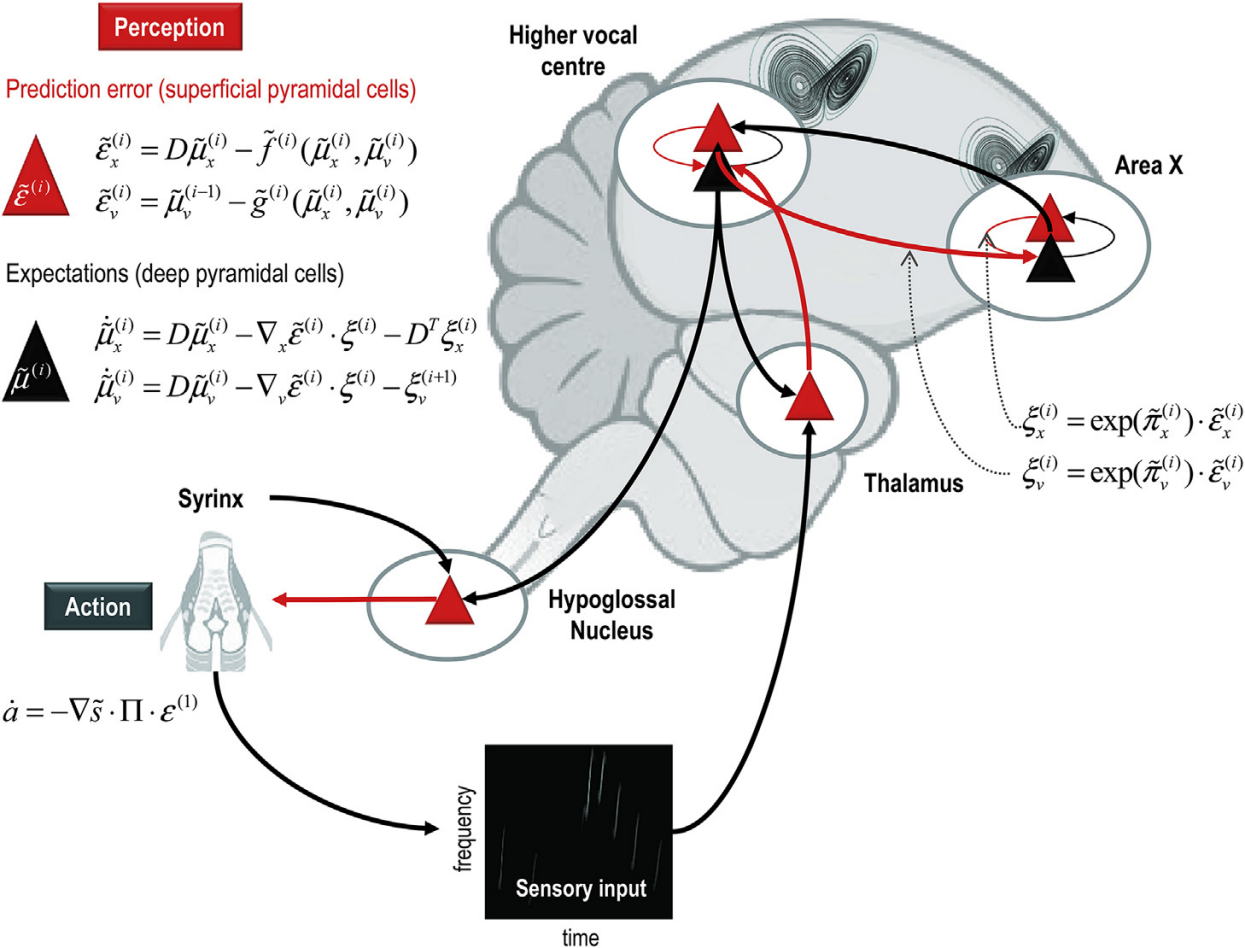
This figure summarizes hierarchical neuronal message passing in predictive coding using the (simplified) neuroanatomy of a songbird. Neuronal activity encodes expectations about the causes of sensory input, where these expectations minimize prediction error. Prediction error is the difference between (ascending) sensory input and (descending) predictions of that input. Here, sensory input is represented by a sonogram encoding the amplitude of different acoustic frequencies over time. Minimising prediction error rests upon recurrent neuronal interactions among different levels of the cortical hierarchy. The available evidence suggests that superficial pyramidal cells (red triangles) compare the expectations (at each level) with top-down predictions from deep pyramidal cells (black triangles) of higher levels. **Left panel**: these equations represent the neuronal dynamics implicit in predictive coding. Prediction errors at the *i*-th level of the hierarchy are simply the difference between the expectations encoded at that level and top-down predictions of those expectations. The expectations *per se* are driven by prediction errors so that they reduce the sum of squared (precision weighted) prediction error. See the appendix for a detailed explanation of these equations and the variables in this figure. **Right panel**: this provides a schematic example in the auditory system of a songbird: it shows the putative cells of origin of ascending or forward connections that convey (precision weighted) prediction errors (red arrows) and descending or backward connections (black arrows) that construct predictions. In this example, area X sends predictions to HVC (c.f., high vocal centre), which projects to the auditory thalamus. However, the HVC also sends proprioceptive predictions to the hypoglossal nucleus, which are passed to the syrinx to generate vocalisation through classical reflexes. These predictions can be regarded as motor commands, while the descending predictions of auditory input correspond to corollary discharge. Note that every top-down prediction is reciprocated with a bottom-up prediction error to ensure predictions are constrained by sensory information. The neuroanatomy implicit in this schematic should not be taken too seriously: we have simply transcribed a generic hierarchical message passing scheme onto the key connections among the cardinal regions implicated in the processing of birdsongs (Nottebohm, 2005).

In summary, predictive coding represents a biologically plausible scheme for updating beliefs about states of the world using sensory samples: see Fig. 2. In this setting, cortical hierarchies are a neuroanatomical embodiment of how sensory signals are generated; for example, a face generates surfaces that generate textures and edges and so on, down to retinal input. This form of hierarchical inference explains a large number of anatomical and physiological facts as reviewed elsewhere (Adams, Shipp, & Friston, 2013; Bastos et al., 2012; Friston, 2008). In brief, it explains the hierarchical nature of cortical connections; the prevalence of backward connections and many of the functional and structural asymmetries in the extrinsic connections that link hierarchical levels (Zeki & Shipp, 1988). These asymmetries include the laminar specificity of forward and backward connections, the prevalence of nonlinear or modulatory backward connections and their spectral characteristics – with fast (e.g., gamma) activity predominating in forward connections and slower (e.g., beta) frequencies that accumulate evidence (prediction errors) ascending from lower levels.

### 2.2 Precision engineered message passing

One can regard ascending prediction errors as broadcasting ‘newsworthy’ information that has yet to be explained by descending predictions. However, the brain has to select the channels it listens to – by adjusting the volume or *gain* of prediction errors that compete to update expectations in higher levels. Computationally, this gain corresponds to the precision or confidence associated with ascending prediction errors. However, to select prediction errors the brain has to estimate and encode their precision (i.e., inverse variance). Having done this, prediction errors can then be weighted by their precision so that only precise information is assimilated at high or deep hierarchical levels. The dynamic and context-sensitive control of precision has been associated with attentional gain control in sensory processing (Feldman & Friston, 2010; Jiang, Summerfield, & Egner, 2013) and has been discussed in terms of affordance in active inference and action selection (Cisek, 2007; Frank, Scheres, & Sherman, 2007; Friston et al., 2012). Crucially, the delicate balance of precision at different hierarchical levels has a profound effect on veridical inference – and may also offer a formal understanding of false inference in psychopathology (Adams, Stephan, Brown, Frith & Friston, 2013; Fletcher & Frith, 2009).

In (Friston and Frith, 2015) we illustrated the role of precision in mediating sensory attenuation and its necessary role in enabling (open loop) motor control during action. See also (Brown, Adams, Parees, Edwards, & Friston, 2013). In brief, this leads to an alternation between sensory attention and attenuation in the action–perception cycle, which involves a temporary suspension of attention to the consequences of acting during the act itself: see (Numminen, Salmelin, & Hari, 1999). We will use the same approach in this paper to model turn taking (Wilson & Wilson, 2005) in the switching between speaking (singing) and listening.

### 2.3 Active inference

Hitherto, we have only considered the role of predictive coding in perception through minimising surprise or prediction errors. However, there is another way to minimise prediction errors; namely, by re-sampling sensory inputs so that they conform to predictions: in other words, changing sensory inputs by changing the world through action. This is known as active inference (Friston, Mattout, & Kilner, 2011). In active inference, action is regarded as the fulfilment of descending proprioceptive predictions by classical reflex arcs. In other words, we believe that we will execute a goal-directed movement and this belief is unpacked hierarchically to provide proprioceptive, and exteroceptive predictions generated from our generative or forward model. These predictions are then fulfilled automatically by minimizing proprioceptive prediction errors at the level of the spinal cord and cranial nerve nuclei: see (Adams, Shipp, et al., 2013) and Fig. 2. Mechanistically, descending proprioceptive predictions provide a target or *set point* for peripheral reflex arcs – that respond by minimising (proprioceptive) prediction errors.

### 2.4 Perception and learning

The preceding aspects of active inference are concerned with expectations about hidden states and causes of sensations and how these expectations minimise prediction error. However, exactly the same arguments apply to the parameters of generative models. Model parameters are quantities that do not change over time and encode causal regularities and associations. Formally, these are the parameters of the functions in Fig. 2. Neurobiologically, the parameters of a generative model are thought to be encoded by synaptic connection strengths and their Bayesian updates look very much like (experience-dependent) associative plasticity that mediates short and long-term changes in synaptic connectivity: see the Appendix and (Friston, 2008) for details. The distinction between states and parameters of generative models induces the distinction between perceptual *inference* and *learning* that proceed over different timescales. Because parameters do not change with time, they accumulate prediction errors over time and are therefore updated at a slower timescale. Equipped with a formal description of perceptual inference and learning, we can now examine the nature of communication using simulations for any given generative model. In the next section, we will apply the computational scheme above to a model of auditory exchange between two systems that are actively trying to infer each other.

## 3 Birdsong and attractors

This section introduces the simulations of birdsong that we will use to illustrate active inference and communication in subsequent sections. The basic idea here is that the environment unfolds as an ordered sequence of states, whose equations of motion induce attractor manifolds that contain sensory trajectories. If we consider the brain has a generative model of these trajectories, then we would expect to see attractors in neuronal dynamics that are trying to predict sensory input. This form of generative model has a number of plausible characteristics:

Models based upon attractors can generate and therefore encode structured sequences of events, as states flow over different parts of the attractor manifold (a subset of states to which the flow is attracted). These sequences can be simple, such as the quasi-periodic attractors of central pattern generators or can exhibit complicated sequences of the sort associated with itinerant dynamics (Breakspear & Stam, 2005; Rabinovich, Huerta, & Laurent, 2008). Furthermore, hierarchically deployed attractors enable the brain to predict or represent sequences of sequences. This is because any low-level attractor embodies a family of trajectories. A natural example here would be language (Jackendoff, 2002). This means it is possible to generate and represent sequences of sequences and, by induction sequences of sequences of sequences etc. This rests upon the states of neuronal attractors at any cortical level providing control parameters for attractor dynamics at the level below (Kiebel, von Kriegstein, Daunizeau, & Friston, 2009). In the example below, we will show how attractor dynamics furnish generative models of sensory input, which behave much like real brains, when measured electrophysiologically.

We first reproduce the simulations reported in (Friston and Frith, 2015) to illustrate the basic nature of the generalised synchrony induced by predictive coding. We then present new results showing that the acquisition of this synchrony emerges naturally, when two predictive coding schemes try to predict each other. This acquisition resolves the hermeneutic problem by underwriting online inference about the causes of shared sensory consequences. A more detailed description of the birdsong model can be found in (Friston & Kiebel, 2009).

### 3.1 A synthetic songbird

**Fig. 3:**
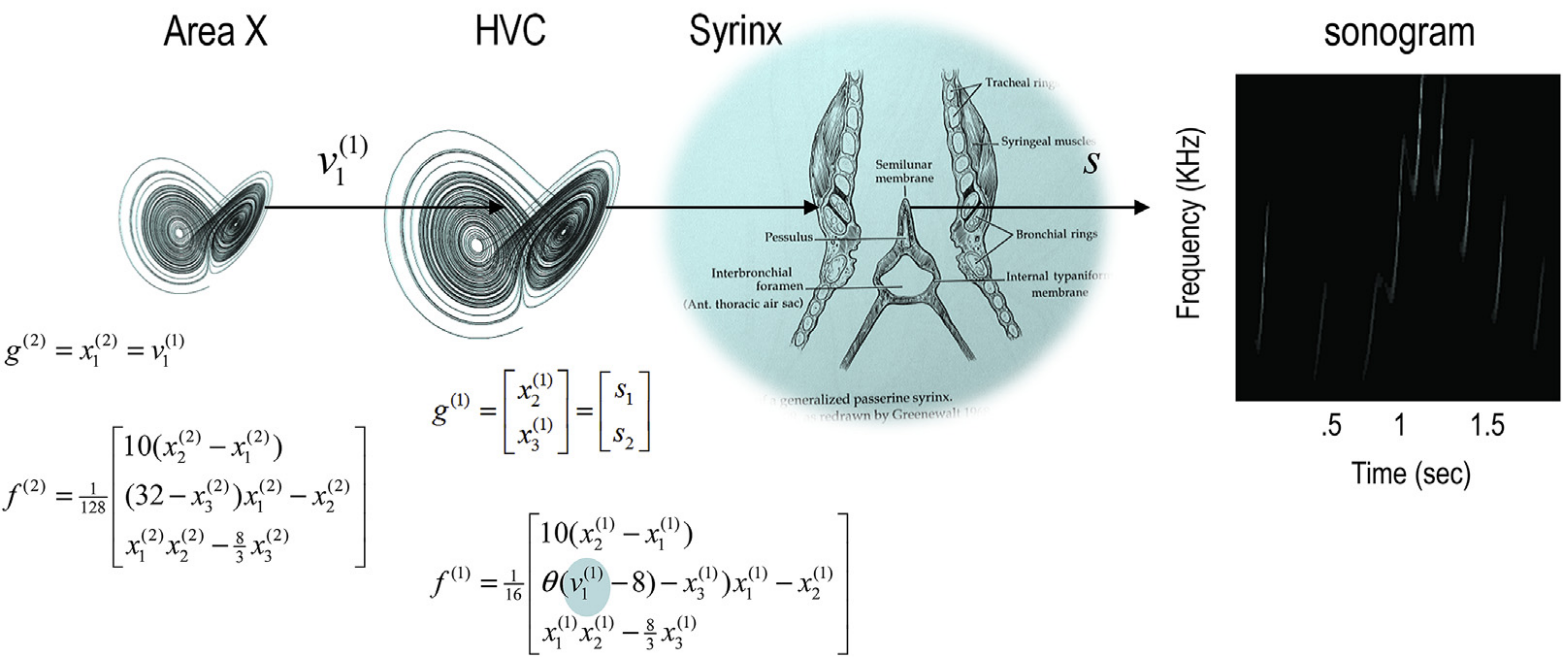
Schematic showing the construction of a generative model for birdsongs. The upper panel illustrates the generative model that comprises two Lorenz attractors, where the higher attractor delivers a control parameter (cyan circle) to a lower level attractor, which, in turn, controls a synthetic syrinx to produce amplitude and frequency modulated stimuli. This stimulus is represented as a sonogram in the right panel. The equations represent the hierarchical dynamic model in the form described in the Appendix.

The example used here deals with the generation and recognition of birdsongs. We imagine that birdsongs are produced by two time-varying states that control the frequency and amplitude of vibrations of the syrinx of a songbird (Fig. 3). There is an extensive modelling effort using attractor models to understand the generation of birdsong at the biomechanical level (Mindlin & Laje, 2005). Here, we use attractors to provide time-varying control over the resulting sonograms. We drive the syrinx with two states of a Lorenz attractor, one controlling the frequency (between two to five KHz) and the other controlling the amplitude or volume. The parameters of the Lorenz attractor were chosen to generate a short sequence of chirps every few hundred milliseconds or so. The Lorenz form for these dynamics is a somewhat arbitrary choice but provides a ubiquitous model of chaotic dynamics in the physical (Poland, 1993) and biological (de Boer & Perelson, 1991) sciences.

**Fig. 4:**
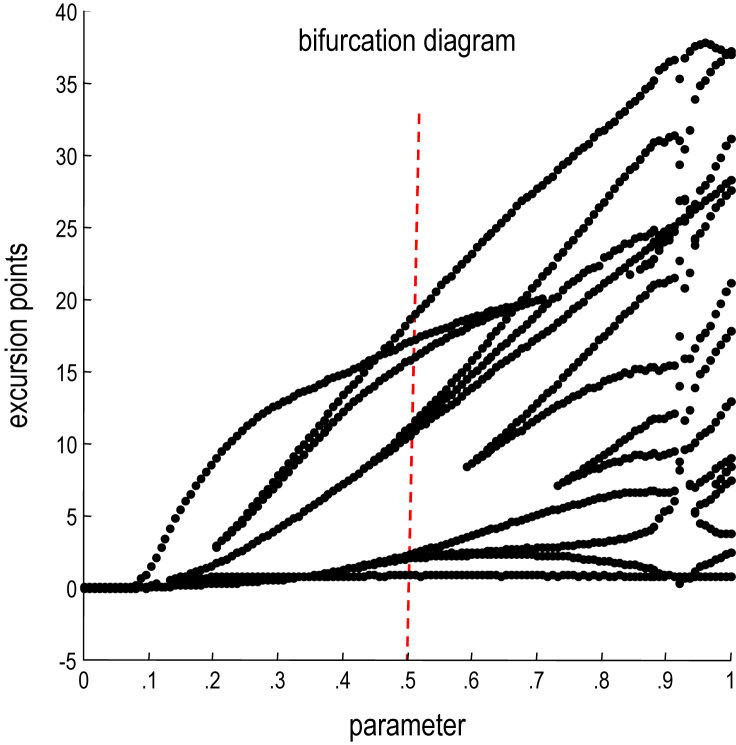
Bifurcation diagram showing the dependency of the dynamics on the model parameter. This diagram plots the maxim and minima of a bird's auditory expectations (of hidden sensory states) over 1 sec of simulated time (while singing to itself). This was repeated for 128 values of the parameter ranging from 0 to 1. The ensuing lines show a succession of bifurcations, indicating the emergence of new peaks or transients in the song trajectory. As the parameter falls to zero, the first level attractor is effectively disconnected from the higher attractor and ceases to show chaotic behaviour. The vertical red line shows the value of the parameter we will use later to illustrate learning.

To give the generative model a hierarchical structure, we placed a second Lorenz attractor, whose dynamics were an order of magnitude slower, over the first. The first state of the slow (extrasensory) attractor provided a control parameter for the fast (sensory) attractor generating the sonogram. In fluid dynamics, this control parameter is known as a *Rayleigh number* and reflects the degree of convective or turbulent flow, which we will associate with dynamical prosody. In other words, the state of the slower attractor changes the manifold of the fast attractor. This manifold could range from a fixed-point attractor, where the states collapse to zero; through to quasi-periodic and chaotic behaviour associated with a high Rayleigh number. Because higher states evolve more slowly, they modulate the chaotic behaviour of the sensory attractor, generating songs, where each song comprises a series of distinct chirps. As shown in Fig. 3, the Rayleigh number linking hierarchical levels depends on a model parameter *θ* that controls the influence of the higher attractor over the lower attractor. High values of this parameter increase the dynamical prosody of the song (inducing successive bifurcations: see Fig. 4). We will use this parameter later to demonstrate perceptual learning and closure of the hermeneutic circle.

### 3.2 Omission and violation of predictions

**Fig. 5:**
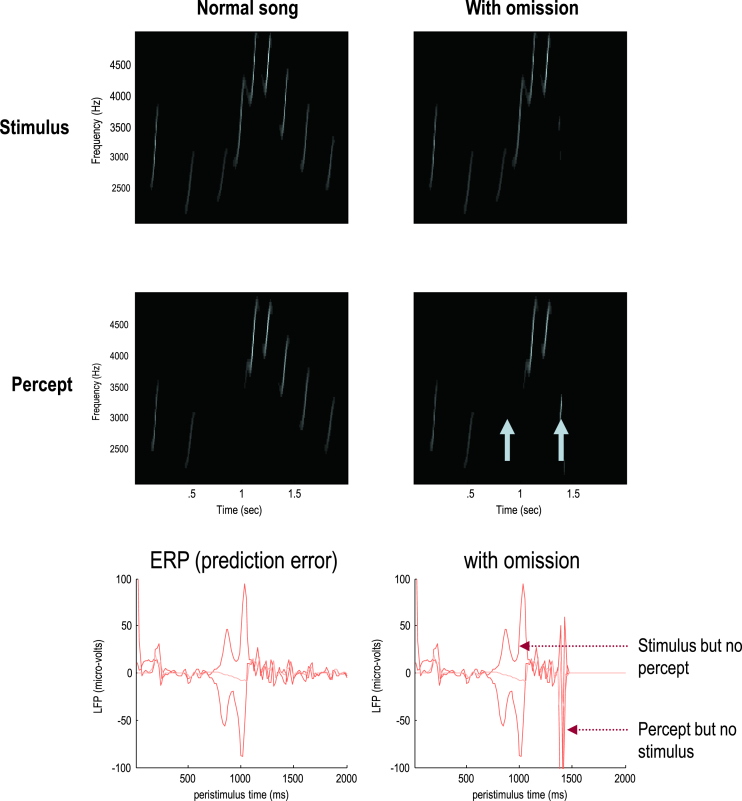
Omission-related responses: The left panels show the original song and responses evoked. The right panels show the equivalent responses to omission of the last chirps. The top panels show the stimulus and the middle panels the corresponding percept in sonogram format. The interesting thing to note here is the occurrence of an anomalous percept after termination of the song on the lower right. This corresponds roughly to the chirp that would have been perceived in the absence of omission. The lower panels show the corresponding (precision weighted) prediction error under the two stimuli at both levels. These show a burst of prediction error when a stimulus is missed and at the point that the stimulus is omitted (at times indicated by the arrows on the sonogram). The solid lines correspond to sensory prediction error and the broken lines correspond to extrasensory prediction error at the second level of the generative model.

To illustrate the predictive nature of predictive coding, perceptual inference was simulated by integrating the above scheme. Formal details of the generative model and integration scheme are provided in the equations in Fig. 3 and the Appendix respectively.^1^ A more detailed description of this simulation can be found in a companion paper (Friston and Frith, 2015). In brief, a sonogram was produced using the above composition of Lorentz attractors (with *θ* = 1) and played to a synthetic bird – who tried to infer the underlying hidden states of the sensory and extrasensory attractors (associated with the HVC and area X respectively). Crucially, we presented two songs to the bird, with and without the final chirps. The corresponding sonograms and percepts (predictions) are shown with their prediction errors in Fig. 5.

The left panels show the stimulus and percept, while the right panels show the stimulus and responses to omission of the last chirps. These results illustrate two important phenomena. First, there is a vigorous expression of prediction error after the song terminates prematurely. This reflects the dynamical nature of inference because, at this point, there is no sensory input to predict. In other words, the prediction error is generated entirely by descending predictions. It can be seen that this prediction error (with a percept but no stimulus) is larger than the prediction error associated with the third and fourth chirps that are not perceived (stimulus but no percept). Second, there is a transient percept when the omitted chirp should have occurred. As noted in (Friston and Frith, 2015), this simulation of omission-related responses as measured with ERPs (Bendixen, SanMiguel, & Schröger, 2012) is particularly interesting, given that non-invasive electromagnetic signals arise largely from superficial pyramidal cells. These are the cells thought to encode prediction error (Bastos et al., 2012).

## 4 A Duet for one

We now turn to the perceptual coupling or communication by simulating two birds that can hear themselves (and each other). Each bird listened for 2 sec, with a low proprioceptive and a high exteroceptive precision (log-precisions of −8 and 2 respectively) and then sang for 2 sec, with high proprioceptive and attenuated auditory precision (log-precisions of 0 and -2 respectively). The log-precisions at the higher level were fixed at 4. Crucially, when one bird was singing the other was listening. This *turn taking* (Wilson & Wilson, 2005) is a natural consequence of active inference because attending to the consequences of action interferes with descending predictions about movement – predictions that are generated before their consequences are evident. There are many examples of the implicit sensory attenuation during self-made acts (Hughes, Desantis, & Waszak, 2013). A physiological illustration can be found in (Agnew, McGettigan, Banks, & Scott, 2013), who show that articulatory movements attenuate auditory responses to speech using fMRI. Furthermore, recent TMS studies suggest that the motor system plays an explicit role in semantic comprehension (Schomers, Kirilina, Weigand, Bajbouj, & Pulvermuller, 2014). We illustrated the importance of sensory attenuation in the context of simulated birdsong in (Friston and Frith, 2015) by showing sensorimotor delays – implicit in articulating a song – destroy the songs dynamical prosody. This means, the birds can only listen or sing but not do both at the same time.

**Fig. 6:**
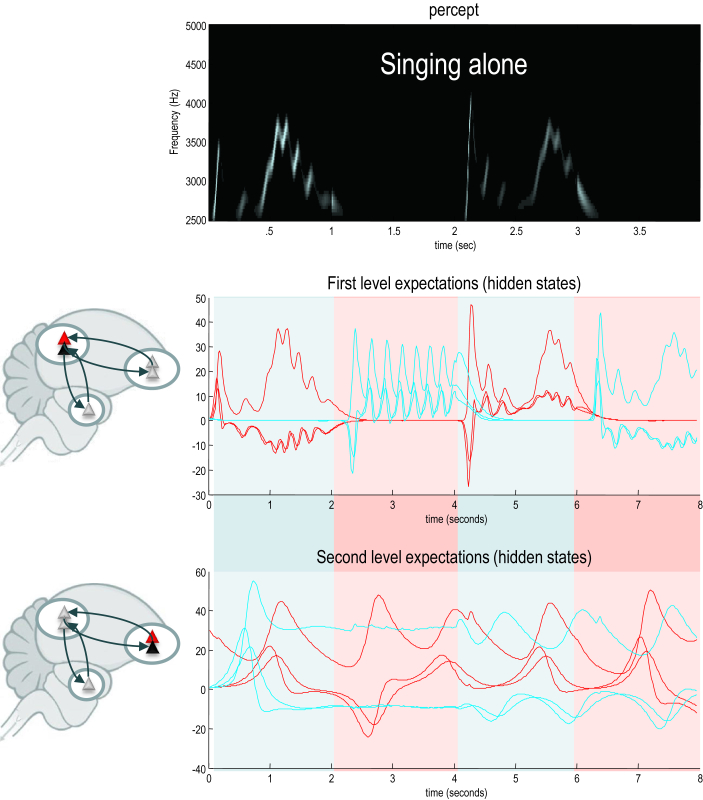
A soliloquy for two. In this simulation, two birds with the same generative models – but different initial expectations – sing for 2 sec and then listen for any response. However, the birds cannot hear each other (because they are too far apart) and the successive epochs of songs diverge due to the sensitivity to initial conditions implicit in these (chaotic) generative models. The upper panel shows the sonogram heard by the first (red) bird. Because this bird can only hear itself, the sonogram reflects the proprioceptive predictions based upon posterior expectations in the HVC (middle panel) and area X (lower panel). The posterior expectations for the first bird are shown in red as a function of time – and the equivalent expectations for the second bird are shown in blue. Note that when the birds are listening, their expectations at the first level fall to zero – because they do not hear anything and auditory input is attended (i.e., has a relatively high precision). This does not destroy the slower dynamics in area X, which is able to generate the song again after the end of each listening period. Note also that the second (blue) bird takes a few hundred milliseconds before it starts singing. This is because it takes a little time for the posterior expectations to find the attractor manifold prescribed by the higher level control parameters.

**Fig. 7:**
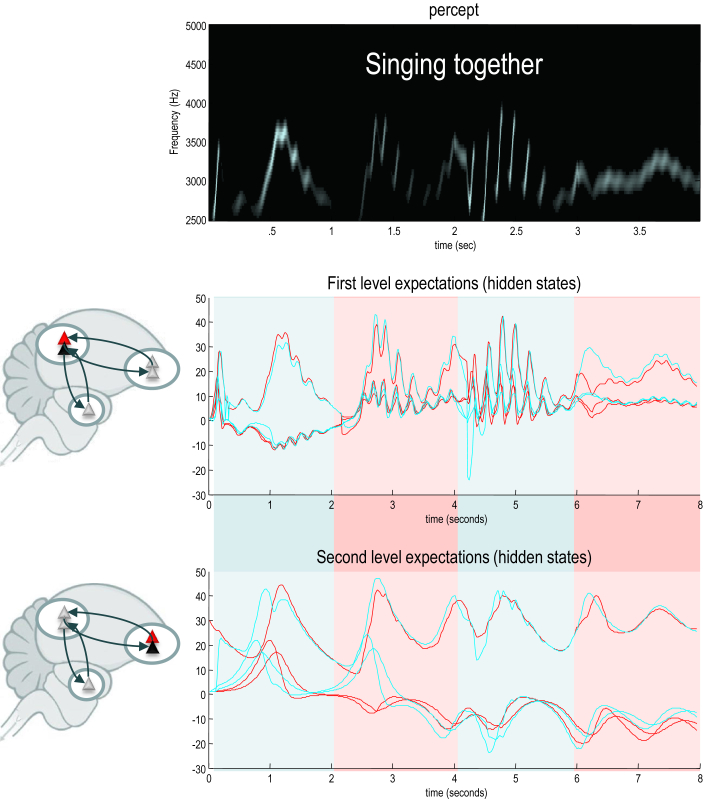
Communication and generalised synchrony. This figure uses the same format as Fig. 6; however, here, we have juxtaposed the two birds so that they can hear each other. In this instance, the posterior expectations show identical synchrony at both the sensory and extrasensory hierarchical levels – as shown in the middle and lower panels respectively. Note that the sonogram is continuous over successive 2 sec epochs – being generated alternately by the first and second bird.

We initialised the simulations with random expectations. This meant that if the birds cannot hear each other, the chaotic dynamics implicit in their generative models causes their expectations to follow divergent trajectories, as shown in Fig. 6. However, if we move the birds within earshot, so that they can hear each other, they synchronise almost immediately. See Fig. 7. This is because the listening bird is immediately entrained by the singing bird to correctly infer the hidden (dynamical) states generating sensations. At the end of the first period of listening, the posterior expectations of both birds approach identical synchrony, which enables the listening bird to take up the song, following on from where the other bird left off. This process has many of the hallmarks of *interactive alignment* in the context of joint action and dialogue (Garrod & Pickering, 2009).

Note that the successive epochs of song are not identical. In other words, the birds are not simply repeating what they have heard – they are pursuing a narrative prescribed by the dynamical attractors (central pattern generators) in their generative models that have been synchronised through sensory exchange. The example in Fig. 7 highlights the fact that the songs articulated by both birds have a rich dynamical vocabulary (including frequency glides, low frequency warbles and amplitude modulated chirps) that is anticipated and reciprocated during the exchange. This means that the birds are singing from the same hymn sheet, preserving sequential and hierarchical structure in their shared narrative. It is this phenomenon – due simply to generalised (in this case identical) synchronisation – that we associate with communication.

**Fig. 8:**
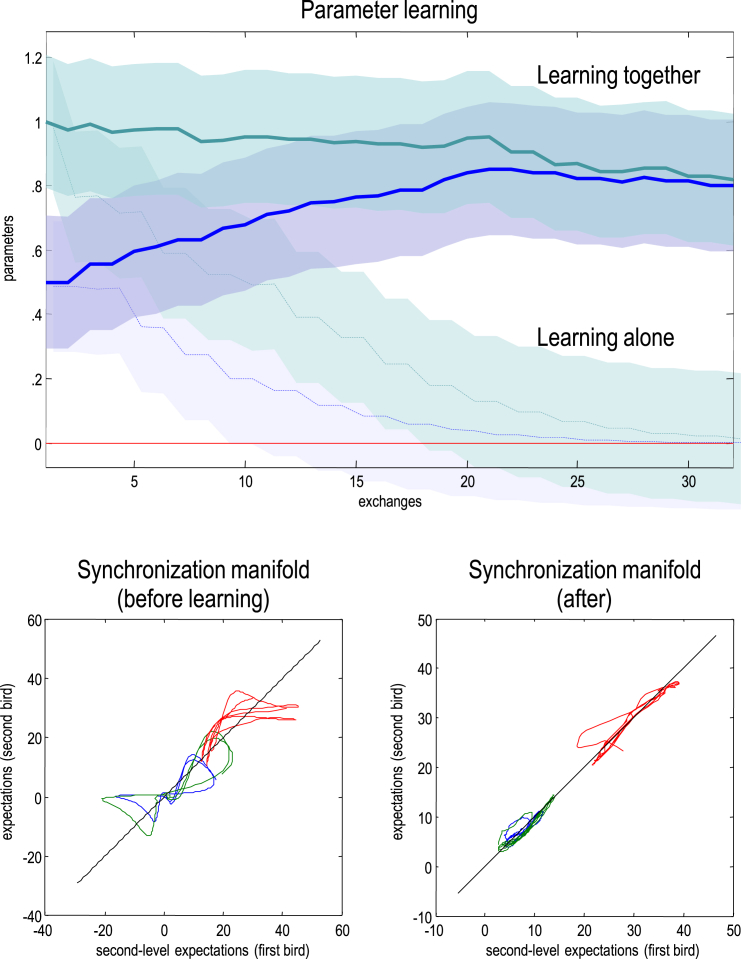
Perceptual learning during communication. Upper panel: this shows epoch by epoch changes in the posterior expectations (lines) of the parameter of the first bird (blue) and second bird (green). The shaded areas correspond to 90% (prior) Bayesian confidence intervals. The broken lines (and intervals) report the results of the same simulation but when the birds could not hear each other. The lower panels show the synchronisation of extrasensory (higher) posterior expectations for the first (left panel) and subsequent (right panel) exchanges respectively. This synchronisation is shown by plotting a mixture of expectations (and their temporal derivatives) from the second bird against the equivalent expectations of the first bird, where this mixture is optimised assuming a linear mapping.

Technically speaking, this simulation of communication shows that two identical dynamical systems – that are predicting each other – necessarily show identical synchronisation. In the language of measure-preserving dynamical systems, generalised synchronisation implies the existence of a random dynamical attractor called a *synchronisation manifold*. The synchronisation manifold is just a set of states to which states are attracted to and thereafter occupy. The simplest example of a synchronisation manifold would be the identity line on a graph plotting homologous states from each system against each other (e.g., the dashed line in Fig. 8). This corresponds to identical synchronisation. Crucially, the attractor (which contains the synchronisation manifold) generally has a low measure or volume. A measure of the attractor's volume is provided by its *measure theoretic* entropy (Sinai, 1959). Although formally distinct from *information theoretic* entropy, both reflect the volume of the attracting set or manifold. This means that minimising prediction errors or free energy (information theoretic entropy) reduces the volume (measure theoretic entropy) of the random dynamical attractor (synchronisation manifold); thereby inducing generalised synchrony. The synchronisation in the example above is identical because both birds share the same generative model. In the next section, we will illustrate generalised synchronisation using simulations were the birds have different models – and how they learn each other's model to produce identical synchronisation.

### 4.1 Summary

In summary, these simulations show that generalised synchrony is an emergent property of coupling active inference systems that are trying to predict each other. It is interesting to consider what is being predicted in this context: the sensations of both birds are simply the consequences of some (hierarchically composed and dynamic) hidden states. But what do these states represent? One might argue that they correspond to some fictive construct that drives the behaviour of one or other bird to produce the sensory consequences that are sampled. But which bird? The sensory consequences are generated, in this setting, by both birds. It therefore seems plausible to assign these hidden states to both birds and treat the agency as a contextual factor (that depends on sensory attention and attenuation). In other words, from the point of view of one bird, the hidden states are amodal, generating proprioceptive and exteroceptive consequences that are inferred in exactly the same way over time; irrespective of whether sensory consequences are generated by itself or another. The agency or source of sensory consequences is determined not by the hidden states *per se* – but by fluctuations in sensory precision that underlie turn taking. In this sense, the expectations are without agency – they are neither yours nor mine, they are *our* expectations.

## 5 Closing the hermeneutic cycle

In this final section, we consider how shared narratives emerge as a natural consequence of perceptual learning driven by, and only by, the minimisation of prediction errors or free energy. Using free energy minimisation, Yildiz, von Kriegstein, and Kiebel (2013) have shown that a hierarchy of nonlinear dynamical systems can learn speech samples rapidly and recognize them robustly, even in adverse conditions. Here, we pursue the same theme but when two birds learn from each other; specifically focussing on sensorimotor learning during which individuals learn to vocalise: see Bolhuis & Gahr (2006) for discussion of birdsong learning. In brief, we repeated the above simulations but reduced the first bird's parameter. This reduces the sensitivity to top-down modulation and suppresses the prosody and richness of the lower attractor. This introduces an asymmetry that precludes identical synchronisation and induces prediction errors that drive learning. This learning corresponds to (epoch by epoch) changes in the posterior expectations of the parameter. In principle, when and only when the parameters are the same is prediction error (free energy) minimised; at which point identical synchronisation should emerge. This effectively closes the hermeneutic cycle to enable precise communication.

Technically, this sort of perceptual learning is a difficult problem and it took us several tries to find a parameterisation that could be learned efficiently. This is because the inference scheme has to estimate unknown parameters in the context of hidden states that also have to be inferred. Furthermore, the generative model is not only dynamical and nonlinear but chaotic. The example we chose is fairly arbitrary but sufficient to show that a biologically plausible inference scheme can solve this class of learning problem. Our particular parameterisation focuses on the link between the sensory and extrasensory attractors and can therefore be regarded as a translation of a narrative into a dynamical vocabulary. This means that our two birds start off with a different mapping between (the same) high-level narrative and the way it is articulated. To understand each other, they have to learn each other's mapping (i.e., vocabulary).

In detail, the first bird's parameter was reduced to *θ* = 0.5, while leaving the second bird's parameter at *θ* = 1. This means the second bird retained a rich prosody, in relation to the first. After each bird sang to the other, their parameters were updated using a simple form of Bayesian belief updating: the prior expectation was replaced by the posterior expectation (retaining a prior precision of 64). The results of this learning are shown in Fig. 8. The upper panel shows the trajectory of the parameter for both birds over 32 (1 sec) exchanges. It can be seen that the parameters of both birds converge towards each other, so that they both come to articulate a relatively rich sequence of songs. With successive exchanges, the parameter of the first bird (blue line) approaches that of the second bird (green line) and synchronisation emerges (lower right panel). The excursions from the synchronisation manifold during the first exchange highlight the initial difficulties the birds have in predicting each other fluently.

This acquisition and maintenance of this rich discourse can be contrasted with the equivalent learning when the birds cannot hear each other. The resulting trajectories are shown as dashed lines and demonstrate that both birds lose the capacity to maintain their Rayleigh number or dynamical prosody. This is because learning occurs predominantly during listening, when the precision of auditory prediction errors (that drive learning) is not attenuated: note the step-like changes in the parameters. However, when they are listening, each bird only hears silence, which is best explained by an attractor with a Rayleigh number of zero (see Fig. 4). In this situation, both birds emit progressively simpler, low-frequency warbles that disappear by about the 16th exchange (results not shown).

Taken together, these simulations illustrate the circular causality inherent in communicative inference and learning. In other words, one needs to infer or predict a structured sensory exchange before learning can occur; while learning is necessary to render sensory exchanges predictable. Furthermore, although the second bird appears to teach the first bird when they hear each other, both are teaching each other – as evidenced by the fact that the second bird ‘forgot’ how to sing when it could not hear the first. The simulations should not be taken as proof of concept of these points; they simply illustrate the sorts of phenomena that can emerge when dynamical systems are trying to predict and learn from each other.

### 5.1 Summary

This section has illustrated perceptual learning as a key process in facilitating communication and generalised synchrony in coupled active inference schemes. In this context, prediction errors implicit in the (neurobiologically plausible) predictive coding implementation of active inference are driving both expectations about hidden states and the learning of model parameters. This means there are two hermeneutic timescales: the first subtending synchronisation and perceptual inference about hidden states of the world and the second slower perceptual learning of the parameters governing fluctuations in the hidden states. The endpoint is a mutual predictability that is underwritten by a convergence of the (parameters of) generative models subtending predictions. Although a simple example, these simulations provide proof of principle that perceptual learning is a sufficient explanation for the emergence of generalised synchrony.

## 6 Conclusion

The treatment above builds on the ideas introduced by (Friston and Frith, 2015); namely, that generalised synchrony – or synchronisation of chaos – provides a formal metaphor for communication and is a natural consequence of active inference. The contribution of the current paper is to show that the same principle (minimisation of free energy or prediction error), also explains the convergent evolution of hierarchical models that generate mutually sympathetic predictions. We have considered this convergence in terms of perceptual learning that closes the hermeneutic cycle.

There are many issues that we have not considered in the domain of neural hermeneutics and communication. Among these is the possibility that we are equipped with multiple generative models that can be deployed depending upon the situation in which we find ourselves, or the person that we are communicating with. In this instance, the selection of an appropriate generative model – that approximates the model selected by you – would be better understood in terms of Bayesian model selection or averaging (FitzGerald, Dolan, & Friston, 2014). This is an interesting possibility that speaks to inferring the context in which we are communicating and who we are communicating with. This represents another (hierarchical) inference problem. The model selection perspective is interesting because, if correct, it implies a multilateral internal model of how we behave that is entirely dependent upon who we are communicating with. In turn, this begs the question of agency and how it is represented in generative models. For example, if our conceptual narratives are context-sensitive and truly shared, am I the same person (in my mind) when speaking to you, as opposed to somebody else? Furthermore, if I do not have a narrative (or appropriate mapping to a vocabulary) that corresponds to your narrative (or mapping), will I ever be able to communicate with you? This is where perceptual learning comes into its own; enabling the acquisition of new narratives (and vocabularies) or repurposing of existing models; e.g., (Adank, Hagoort, & Bekkering, 2010; Skoe, Krizman, Spitzer, & Kraus, 2014). However – as anyone who has tried to learn a foreign language can testify – learning can be a difficult and slow process, especially for Englishmen like us.

## Conflict of interest statement

The authors declare no conflicts of interest.

## Appendix

This brief description of generalized predictive coding is based on (Feldman & Friston, 2010). A more technical description can be found in (Friston, Stephan, Li, & Daunizeau, 2010). This scheme is based on three assumptions:

• The brain minimizes a free energy of sensory inputs defined by a generative model.
• The generative model used by the brain is hierarchical, nonlinear and dynamic.
• Neuronal firing rates encode the expected state of the world, under this model.

Free energy is a quantity from statistics that measures the quality of a model in terms of the probability that it could have generated observed outcomes. This means that minimizing free energy maximizes the Bayesian evidence for the generative model. The second assumption is motivated by noting that the world is both dynamic and nonlinear and that hierarchical causal structure emerges inevitably from a separation of spatial and temporal scales. The final assumption is the Laplace assumption that leads to a simple and flexible neuronal code.

Given these assumptions, one can simulate a whole variety of neuronal processes by specifying the particular equations that constitute the brain's generative model. In brief, these simulations use differential equations that minimize the free energy of sensory input using a generalized gradient descent.

A.1 $$\dot{\tilde{\mu }}\left(t\right)=D\tilde{\mu }\left(t\right)-\partial_{\tilde{\mu }}F\left(\tilde{s}\left(t\right),\tilde{\mu }\left(t\right)\right)$$

These differential equations say that neuronal activity encoding posterior expectations about (generalized) hidden states of the world $\tilde{\mu }=\left(\mu ,\mu^{\prime },\mu^{\prime \prime },\ldots \right)$ reduce free energy – where free energy $F\left(\tilde{s},\tilde{\mu }\right)$ is a function of sensory inputs $\tilde{s}=\left(s,s^{\prime },s^{\prime \prime },\ldots \right)$ and neuronal activity. This is known as generalized predictive coding or Bayesian filtering. The first term is a prediction based upon a differential matrix operator *D* that returns the generalized motion of expected hidden states $D\tilde{\mu }=\left(\mu^{\prime },\mu^{\prime \prime },\mu^{\prime \prime \prime },\ldots \right)$. The second (correction) term is usually expressed as a mixture of prediction errors that ensures the changes in posterior expectations are Bayes-optimal predictions about hidden states of the world. To perform neuronal simulations under this scheme, it is only necessary to integrate or solve Equation A.1 to simulate the neuronal dynamics that encode posterior expectations. Posterior expectations depend upon the brain's generative model of the world, which we assume has the following hierarchical form:

A.2 $$\begin{array}{c} s=g^{\left(1\right)}\left(x^{\left(1\right)},v^{\left(1\right)}\right)+\exp\left(-\frac{1}{2}\pi_{v}^{\left(1\right)}\right)\cdot \omega_{v}^{\left(1\right)}\\ {\dot{x}}^{\left(1\right)}=f^{\left(1\right)}\left(x^{\left(1\right)},v^{\left(1\right)}\right)+\exp\left(-\frac{1}{2}\pi_{x}^{\left(1\right)}\right)\cdot \omega_{x}^{\left(1\right)}\\ \vdots \\ v^{\left(i-1\right)}=g^{\left(i\right)}\left(x^{\left(i\right)},v^{\left(i\right)}\right)+\exp\left(-\frac{1}{2}\pi_{v}^{\left(i\right)}\right)\cdot \omega_{v}^{\left(i\right)}\\ {\dot{x}}^{\left(1\right)}=f^{\left(i\right)}\left(x^{\left(i\right)},v^{\left(i\right)}\right)+\exp\left(-\frac{1}{2}\pi_{x}^{\left(i\right)}\right)\cdot \omega_{x}^{\left(i\right)}\\ \vdots \end{array}$$

This equation describes a probability density over the sensory and hidden states that generate sensory input. Here, the hidden states have been divided into hidden states and causes (*x*^(*i*)^, *v*^(*i*)^) at the *i-*th level within the hierarchical model. Hidden states and causes are abstract variables that the brain uses to explain or predict sensations – like the motion of an object in the field of view.

In these models, hidden causes link hierarchical levels, whereas hidden states link dynamics over time. Here, (*f*^(*i*)^, *g*^(*i*)^) are nonlinear functions of hidden states and causes that generate hidden causes for the level below and – at the lowest level – sensory inputs. Random fluctuations in the motion of hidden states and causes $\left(\omega_{x}^{\left(i\right)},\;\omega_{v}^{\left(i\right)}\right)$ enter each level of the hierarchy. Gaussian assumptions about these random fluctuations make the model probabilistic. They play the role of sensory noise at the first level and induce uncertainty at higher levels. The amplitudes of these random fluctuations are quantified by their log-precisions $\left(\pi_{x}^{\left(i\right)},\pi_{v}^{\left(i\right)}\right)$.

Given the form of the generative model (A.2) we can now write down the differential equations (A.1) describing neuronal dynamics in terms of (precision-weighted) prediction errors. These errors represent the difference between posterior expectations and predicted values, under the generative model (using $A\cdot B≜A^{T}B$ and omitting higher-order terms):

A.3 $$\begin{array}{l} {\dot{\tilde{\mu }}}_{x}^{\left(i\right)}=D{\tilde{\mu }}_{x}^{\left(i\right)}+\frac{\partial {\tilde{g}}^{\left(i\right)}}{\partial {\tilde{\mu }}_{x}^{\left(i\right)}}\cdot \xi_{v}^{\left(i\right)}+\frac{\partial {\tilde{f}}^{\left(i\right)}}{\partial {\tilde{\mu }}_{x}^{\left(i\right)}}\cdot \xi_{x}^{\left(i\right)}-D^{T}\xi_{x}^{\left(i\right)}\\ {\dot{\tilde{\mu }}}_{v}^{\left(i\right)}=D{\tilde{\mu }}_{v}^{\left(i\right)}+\frac{\partial {\tilde{g}}^{\left(i\right)}}{\partial {\tilde{\mu }}_{v}^{\left(i\right)}}\cdot \xi_{v}^{\left(i\right)}+\frac{\partial {\tilde{f}}^{\left(i\right)}}{\partial {\tilde{\mu }}_{x}^{\left(i\right)}}\cdot \xi_{x}^{\left(i\right)}-\xi_{v}^{\left(i+1\right)}\\ \xi_{x}^{\left(i\right)}=\exp\left({\tilde{\pi }}_{x}^{\left(i\right)}\right)\cdot {\tilde{\varepsilon }}_{x}^{\left(i\right)}\\ \xi_{v}^{\left(i\right)}=\exp\left({\tilde{\pi }}_{v}^{\left(i\right)}\right)\cdot {\tilde{\varepsilon }}_{v}^{\left(i\right)}\\ {\tilde{\varepsilon }}_{x}^{\left(i\right)}=D{\tilde{\mu }}_{x}^{\left(i\right)}-{\tilde{f}}^{\left(i\right)}\left({\tilde{\mu }}_{x}^{\left(i\right)},{\tilde{\mu }}_{v}^{\left(i\right)}\right)\\ {\tilde{\varepsilon }}_{v}^{\left(i\right)}={\tilde{\mu }}_{v}^{\left(i-1\right)}-{\tilde{g}}^{\left(i\right)}\left({\tilde{\mu }}_{x}^{\left(i\right)},{\tilde{\mu }}_{v}^{\left(i\right)}\right) \end{array}$$

This produces a relatively simple update scheme, in which posterior expectations ${\tilde{\mu }}^{\left(i\right)}$ are driven by a mixture of prediction errors ${\tilde{\varepsilon }}^{\left(i\right)}$ that are defined by the equations of the generative model.

In neuronal network terms, Equation A.3 says that error-units compute the difference between expectations at one level and predictions from the level above (where *ξ*^(*i*)^ are precision weighted prediction errors at the *i*-th level of the hierarchy). Conversely, posterior expectations are driven by prediction errors from the same level and the level below. These constitute bottom-up and lateral messages that drive posterior expectations towards a better prediction to reduce the prediction error in the level below. In neurobiological implementations of this scheme, the sources of bottom-up prediction errors are generally thought to be superficial pyramidal cells, because they send forward (ascending) connections to higher cortical areas. Conversely, predictions are thought to be conveyed from deep pyramidal cells by backward (descending) connections, to target the superficial pyramidal cells encoding prediction error (Bastos et al., 2012; Mumford, 1992).

The corresponding updates for the parameters follow a similar scheme; however, because parameters are time-invariant we have a simpler gradient descent that effectively accumulates free energy gradients over time:

A.4 $$\begin{array}{c} {\dot{\tilde{\mu }}}_{\theta }\left(t\right)=-\partial_{\tilde{\mu }}S\left(\tilde{s},\tilde{\mu }\right)\\ \partial_{t}S=F\left(\tilde{s}\left(t\right),{\tilde{\mu }}_{x}\left(t\right),{\tilde{\mu }}_{v}\left(t\right),{\tilde{\mu }}_{\theta }\right) \end{array}$$

Here, $S\left(\tilde{s},\tilde{\mu }\right)$ is the anti-derivative of free energy (known as free action). Neurobiologically, when expressed in terms of prediction errors, the solution to Equation A.4 corresponds to associative plasticity. See (Friston, 2008) for details.

## Footnotes

1 For people interested in the technical details – and exploring the effects of changing parameters – the routines generating the figures in this paper are available as part of the academic SPM freeware (http://www.fil.ion.ucl.ac.uk/spm/). Annotated Matlab routines can be run and edited by invoking a graphical user interface (by typing **DEM**) and selecting **Birdsong duet (DEM_demo_duet.m)**.
